# Published Articles in PubMed-indexed Journals from Tabriz University of Medical Sciences Faculty of Dentistry

**DOI:** 10.5681/joddd.2012.033

**Published:** 2012-12-11

**Authors:** Negin Ghasemi, Saeed Rahimi, Shahriar Shahi, Hadi Mokhtari

**Affiliations:** ^1^Post-graduate Student, Department of Endodontics, Faculty of Dentistry, Tabriz University Medical Science, Tabriz, Iran; ^2^Dental and Periodontal Research Center, Tabriz University of Medical Science, Tabriz, Iran; ^3^Professor, Department of Endodontics, Faculty of Dentistry, Tabriz University of Medical Science, Tabriz, Iran; ^4^Assisstant Professor, Department of Endodontics, Faculty of Dentistry, Tabriz University of Medical Science, Tabriz, Iran

**Keywords:** Dental, faculty, medical, scientific publication, university

## Abstract

**Background and aims:**

This survey was conducted to provide statistical data regarding publications in PubMed-indexed journals from Tabriz University of Medical Sciences Faculty of Dentistry.

**Materials and methods:**

The database used for this study was PubMed. The search was conducted using key words including the names of the heads of the departments. Papers published between January 1, 2005 and April 31, 2012 were considered. The retrieved abstracts were reviewed and unrelated articles were excluded. Data were transferred to Microsoft Excel software for descriptive statistical analyses.

**Results:**

A total of 158 papers matched the inclusion criteria, with the majority from the Department of Endodontics (49 articles). The highest proportion (48.3%) of papers was related to in vitro studies, followed by clinical trials, in vivo studies, and case reports. The number of publications showed a considerable increase over the studied period.

**Conclusion:**

PubMed-indexed publications from different departments have increased steadily, suggesting that research has become an essential component in the evaluated institute.

## Introduction

It is obvious that research is an important aspect of health care service. Research publications are good indicators for determining the extent and quality of research in a given institute or territory.^1-3^ In order to assess the number of papers published in medical journals, the database of PubMed is a valuable resource. The search engine can be used as a means to obtain data for analysis. In addition, the data can reflect the community performance in research progress and level of contribution to global sciences and significantly influences future planning in research.^4^

As there is no comprehensive data that can show the performance of Tabriz University of Medical Sciences Faculty of Dentistry in research since it was established in 1987, this evaluation was designed to assess the research output of this educational center in PubMed-indexed journals over a period of recent eight years.

## Materials and Methods

Data regarding publications from Tabriz University of Medical Sciences Faculty of Dentistry were retrieved from PubMed. For data collection, the search was conducted using key words including the names of the heads of the departments of the Faculty of Dentistry. Only papers published between January 1, 2005 and April 31, 2012 were considered. To avoid false positives, the abstracts of all the retrieved articles published during that period were carefully screened and unrelated articles that did not match the following inclusion criteria were excluded: (1) study conducted in the Faculty of Dentistry and the article submitted from the same institute; (2) authors working in the Faculty of Dentistry at the at the time of paper submission and publication; (3) study conducted in Tabriz Faculty of Dentistry but at the time of publication the authors were working out of the Faculty; (4) study from other university in Iran (according to the first author’s affiliation), with one of the authors from Tabriz Faculty of Dentistry.

The first author’s name and affiliation as well as those of the other authors, the type of the published paper, and the year of publication were recorded for all papers.

Data were transferred to Microsoft Excel software for descriptive statistical analyses.

## Results

In this study, 158 papers published during the defined eight-year period matched the inclusion criteria. Figure 1 shows the year-wise distribution of publications from 2005 to 2012. The number of articles showed a steady increase over the eight-year period.

Figure 1. Year-wise distribution of the publications (a) and the distribution of the type of the published papers (b) in the evaluated eight-year period.

**a:**
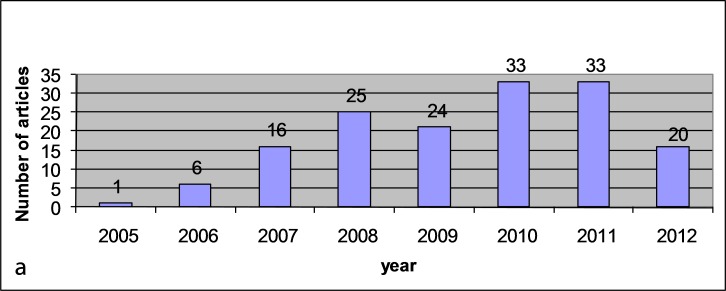

**b:**
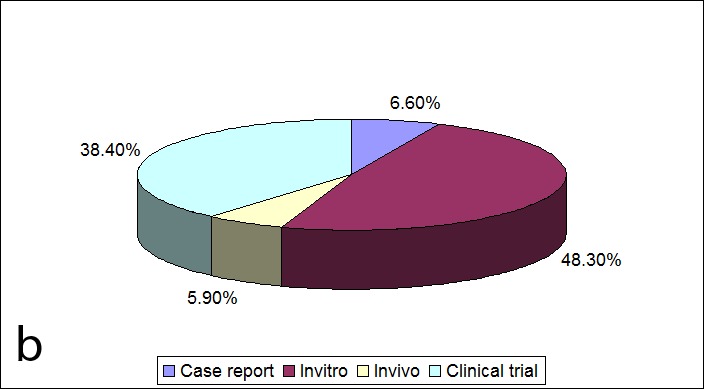

Distribution of the type of the published studies is also presented in Figure 1. The highest proportion (48.3%) of papers was related to in vitro studies, followed by clinical trials, in vivo studies, and case reports.

Comparing the number of papers related to each department, the highest was found to be of the Department of Endodontics (49 papers; Table 1).

**Table 1:** Ranking of departments regarding published articles in PubMed-indexed Journals

Department	Number of articles
Endodontics	49
Operative dentistry	32
Pediatric dentistry	27
Oral and maxillofacial surgery	21
Periodontics	20
Oral pathology	10
Orthodontics	9
Prosthodontics	8
Oral medicine	7
Community dentistry	6
Oral Radiology	1

## Discussion

Scientific publications are considered the best source for providing new information in each academic discipline. Due to the growing complexity of science and technology, reliable indicators should be devised for all aspects of scientific development.^1,4^ Despite their many imperfections, indicators of science and technology are key elements of any global overview as they offer a means by which we might monitor and compare countries and regions of varying geographic sizes and socio-economic development.^2,4^ PubMed is a comprehensive database run by the US National Library of Medicine,^1,3^ and was, therefore, used for retrieving the required data related to the publications the Faculty of Dentistry in this study.

Assessing the number of published papers from a given educational center can be a reliable indicator to evaluate scientific performance. This is the first study assessing the publications in PubMed-indexed journals from Tabriz University of Medical Sciences Faculty of Dentistry. Based on careful scrutiny of all the publications as detailed in the methods, it seems reasonable to conclude that the current study presents near-accurate data regarding the subject.

The results demonstrated that the number of papers in PubMed-indexed journals from the Faculty of Dentistry has steadily increased over the studied eight-year period. The majority of the articles were related to in vitro studies and clinical trial trials. Based on the hierarchy in evidence-based dentistry, meta-analyses, systematic reviews, and randomized controlled trials (RCT) rank as the first-quality articles. In the present data, the majority of RCTs were from the Department of Pediatric Dentistry.

An important limitation of the present study in assessing the scientific progression was missing of a considerable number of articles from this institution’s authors not published in PubMed-indexed journals, which were not included in the study.

## Conclusion

The results indicated rapid development of research in the Tabriz University of Medical Sciences Faculty of Dentistry.
